# Uptake of cancer screenings among a multiethnic refugee population in North Texas, 2014-2018

**DOI:** 10.1371/journal.pone.0230675

**Published:** 2020-03-30

**Authors:** Amy Raines Milenkov, Martha Felini, Eva Baker, Rushil Acharya, Elvis Longanga Diese, Sara Onsa, Shane Fernando, Holy Chor

**Affiliations:** 1 Department of Pediatrics & Women’s Health, University of North Texas Health Science Center, Fort Worth, TX, United States of America; 2 Texas College of Osteopathic Medicine, University of North Texas Health Science Center, Fort Worth, TX, United States of America; Anglia Ruskin University, UNITED KINGDOM

## Abstract

**Background:**

Refugees are less likely than US born populations to receive cancer screenings. Building Bridges is a community health worker prevention program designed to increase refugee’s cancer screening uptake. The purpose of this cross sectional analysis was to assess differences in uptake of cervical, breast, liver, and colorectal screens across six cultural groups.

**Methods:**

Data was abstracted in 2018 for this analysis. Participants were categorized into six cultural groups (Myanmar, Central Africa, Bhutan, Somalia, Arabic Speaking Countries, and Other) to assess differences in sociodemographic measures and screening uptake. Uptake proportions were calculated for each cancer type (cervical, breast, liver, and colon) among eligible participants, by gender and cultural group. Differences in uptake across groups were assessed using stratified analysis and logistic regression. Prevalence odds ratios (POR) and 95% confidence intervals (CIs) were calculated for each group to assess the association between screening completion and cultural group.

**Findings:**

874 refugees were asked about cancer screening history. The majority of participants were either ‘never had been screened’ or ‘not up-to-date’ for every cancer screening. Among age eligible, 82% had no prior pap exam within the past 3 years, 81% had no prior mammogram within the past year, 69% didn’t know their Hepatitis B status and 87% never had a colon cancer screening. Overall, higher uptake of all types of cancer screenings was observed in Myanmar and Bhutanese groups, except colon cancer screening which was higher among Central African Region and Arabic Speaking participants.

**Conclusion:**

Screening uptake varied by ethnic group and screening type. The program reached an under and never screened population, however, the proportion of refugees who received a cancer screening remained low compared to the US population. Diversity within refugee communities requires adaptation to specific cultural and linguistic needs to include new Americans in cancer elimination efforts.

## Introduction

Despite recent changes in refugee resettlement policy, the United States (U.S.) remains the top refugee resettlement country in the world. [1] The U.S. has admitted over 3.3 million refugees since 1975. [2] During the 2016 fiscal year alone, 84,995 refugees resettled in the U.S. with most arriving from the Democratic Republic of the Congo, Burma, Iraq, and Somalia. Between 2002 and 2017, Texas was the second refugee resettlement destination after California. [3]

Most of the countries of origin of recent refugees are in the midst of an epidemiologic transition characterized by a decrease in infectious diseases and an increase in the incidence of cancers and other non-communicable diseases. [4–6] Cancer prevention services in these low-income countries, however, are either nonexistent or inaccessible for the majority of the population. [7,8] Refugees represent a specific sub-category of immigrants to the U.S. The universal definition of a refugee according to the 1951 Convention Relating to the Status of Refugees contained in Article 1(A)(2) of the 1951 Convention and 1967 Protocol Amendment is someone, “owning to a well-founded fear of being persecuted for reasons of race, religion, nationality, membership of a particular social group or political opinion, is outside the country of his nationality and is unable or owing to such fear, is unwilling to avail himself of the protection of that country; or who, not having a nationality and being outside the country of his former habitual residence, is unable or, owing to such fear, is unwilling to return to it.” [9]

Refugees face multiple challenges following resettlement in the U.S. that may contribute to lower cancer screenings. These challenges include language and cultural barriers, fear of procedures, history of trauma, work schedules, childcare commitments, and a lack of understanding the U.S. medical infrastructure. [10–17] Previous studies found refugees are less likely to get screened for colon, cervical, and breast cancers compared to U.S.-born populations. These studies, however, are generally small and without a comparison of screening uptake with other refugee groups. [18–21]

The purpose of this cross-sectional study was to assess differences in uptake of cervical, breast, liver, and colorectal screens across six immigrant groups participating in the Building Bridges program. Identifying differences in demographic and screening behaviors and acceptance among different refugee groups may help clinicians and cancer prevention program planners recognize the need for culturally sensitive and individually tailored approaches between the ethnic groups that make up the broader refugee community, and thus reduce the cancer burden among these groups.

## Methods

### Overview of Building Bridges program protocols

The Building Bridges Program started in 2014 using a community health worker model to engage and provide refugees residing in Fort Worth, Texas, with culturally and linguistically appropriate cancer prevention education. For those eligible, a subsequent opportunity to participate in a breast, cervical, liver, and/or colon cancer screening was provided. Refugees representing the greatest number of arrivals to Texas were engaged for the community program. The primary groups engaged included ethnic subgroups from Myanmar, and Central African region, Bhutanese, Somali and Sudanese immigrants.

Community health workers (CHWs) from each of the primary groups were recruited during the design phase of the program as full-time, benefit eligible employees of the university. CHWs were recommended by community leaders as helpful, bilingual (English speaking), community members. Some had previous case work, counseling, and public health experience. Each CHW received training in cancer prevention, causes and treatment, preventive screenings, overall health and wellness, health care systems, case management procedures, data collection, medical interpretation and related topics as part of their initial training and throughout the duration of the community project. Videos, anatomical models, presentations, expert speakers, and field trips were all used in training.

CHWs actively recruited members from their community during religious, community, education events, (eg. English as a Second Language classes), as well as in community locations where resettlement agencies initially resettle refugees. Each CHW used recruitment methods they felt most comfortable with and effective in their cultures. Additionally, they used their own social networks and encouraged “word-of-mouth” recruitment, whereby participants refer others to the program. Those who wanted to enroll in the program provided their phone numbers to CHWs at outreach events, and CHWs followed up to schedule one-on-one enrollments in client’s home.

Once enrolled, participants were provided with culturally appropriate cancer prevention educations in each of the four cancers by the trained bicultural and bilingual community health workers from seven refugee communities/regions and language group (i.e., Bhutanese, Karen, Chin, Somali, Arabic speaking, Nepali, and Central African region). Education sessions were conducted in 30 different languages and occurred in both individual and group settings. Eligibility for program participation included: 18 years of age or older and immigrated to the U.S. as a refugee or asylee, or member of an immigrant group that came to the U.S. primarily as refugees. Education was made available to any interested community member meeting the program eligibility, regardless of eligibility for cancer screenings.

The education program was adapted from the National Cancer Institute’s Research-Tested Intervention Programs (RTIPs) [22] to be applicable to respective ethnic groups. Education tools included videos, presentations and the use of anatomical models. Each education was adapted for the targeted community with regards to language, culture, learning styles and literacy levels. CHWs and community leaders from the different communities assisted in every phase of the adaptation process, including translation and field testing of consents, assessments and study documents.

Part of the education process was completing a baseline assessment which was administered by CHWs. Participants self-reported information when asked by CHW on demographics (gender, marital status, age, country of origin, and family size), social determinants (healthcare services, insurance status, employment status, education level, and language literacy), migration history (age at U.S. entry, time in the U.S. at enrollment), and previous cancer screening history. CHWs briefly described cancer screening procedures before participants reported on cancer screening history to reduce recall errors.

After the education, participants were provided an opportunity to participate in breast, cervical, liver, and/or colon cancer screening. Eligibility for cancer screenings was determined by age, gender and the date of last exam according to the U.S. Department of Health and Human Services U.S. Preventive Services Task Force’s Cancer Screening Guidelines.[23] That is, women aged 21 and older who responded, “never had pap exam in the past” or “not within the last three years” of their enrollment in our program or responded “I don’t know” were eligible for cervical cancer screening. Similarly, women aged 40 and older who responded “never”, or “I don’t know”, or “not within the previous 12 months” when asked for previous history of mammogram were considered to be eligible for breast cancer screening. All participants who could not recall their Hepatitis B status from their post-arrival medical screening conducted for all newly arrived refugees in the United States were offered Hepatitis B screening. Colorectal screenings (colonoscopy and/or fecal immunochemical test, (FIT) for participants aged 50 and older were added in 2017 as part of a CPRIT funded expansion grant. Participants were offered colorectal screenings if they responded they had not had one in the past year, or never had a screening. Cancer screenings included ThinPrep Pap Test, mammograms, Hepatitis B Virus (HBV) blood panel, and the FIT and/or colonoscopy. Clinics sent cervical, breast, and colon screening results directly to the participant. CHWs followed up with participants to ensure screening results were received (by phone, in person, or letter) and understood to assist with scheduling for diagnostic screenings if needed. Hepatitis B screening results were provided directly to investigators and reviewed with participants by CHWs. Hepatitis B and HPV vaccines were also made available to eligible participants. CHWs assisted participants with health insurance applications, scheduling appointments, arranging for transportation to and from cancer screening sites, coordinating interpretation services, and helping navigate screen positives to diagnostic screenings for follow-up and treatment. Duration of these services depended on the willingness to participate in education and screening, as well as results that needed follow-up services.

Participants self-reported the following countries of origin: Myanmar, Thailand, Democratic Republic of Congo, Rwanda, Burundi, Uganda, Somalia, Kenya, Bhutan, Nepal, Sudan, Iraq, Syria, Egypt, Afghanistan, Angola, Chad, Eritrea, Ethiopia, Jordan, Liberia, Senegal, and Tanzania. For purposes of this cross-sectional analysis, participants were categorized into six major groups by language and geography: (1) Myanmar/Thailand, (2) Central African Region (Democratic Republic of Congo, Rwanda, Burundi, Tanzania, and Uganda), (3) Bhutan/Nepal, (4) Somalia/Kenya, (5) Arabic (Sudan, Iraq, Syria, Egypt, Jordan), and (6) Other (Afghanistan, Angola, Chad, Eritrea, Ethiopia, Liberia, and Senegal).

## Measurements

Data collected prospectively by CHWs throughout the BB Program (2014–2018) was abstracted by investigators in a secondary analysis to assess differences in screening uptake across all cultural groups participating in the BB Program.

### Self-reported baseline measures

Country of origin, gender, age, years lived in the U.S., number of biological children, years of education, fluency in English, employment status, and health insurance status were extracted from baseline assessments for analysis. Formal education was defined as the total number of school years completed in home country and/or in the U.S. Time in U.S. was calculated as time between participants' self-reported arrival date in the U.S. and BB Program enrollment date. History of ever having prior cancer screenings (cervical, breast, liver, and colon) was also abstracted for analysis. When asked ‘Have you ever had a cancer screening before?' participants who responded ‘No', or ‘I don't know' were defined as never screened.

### Screening completion and results

Screening completion dates were abstracted from a ‘BB Program Participant Appointment Tracking Form’, which was maintained by the CHW for tracking purposes. Screening results were abstracted from ‘Building Bridges Appointment Outcome Log’. An abnormal pap screen result was defined as including ASC-US, LSIL, or HSIL. Mammogram results were recorded using the Breast Imaging Reporting and Data System (BI-RADS). Abnormal mammogram result was defined as BI-RAD score 4–6 (4-Suspicious abnormality, 5-Highly suggestive of malignancy, 6-Known biopsy-proven malignancy) and ‘Abnormal result-score unknown’. Results with BI-RAD score 0–3 (0-Additional imaging required, 1-Negative, 2-Benign finding, 3-Probably benign finding) were considered as normal. The HBV results were defined as abnormal if the surface antigen was detected in blood and normal if the surface antigen was negative, regardless of the status of antibodies. For colon cancer screenings, negative FIT results were defined as normal and positive FIT results were defined as abnormal.

## Statistical analysis

Descriptive statistics of baseline measures were calculated across cultural groups. Uptake proportions were calculated for each cancer type (cervical, breast, liver, and colon) among age-eligible participants, by gender and by each cultural group. Differences in uptake of cancer screening services across cultural groups were assessed using stratified analysis and logistic regression. Prevalence odds ratios (POR) and 95% confidence intervals (CIs) were calculated for each cultural group to assess the association between screening completion (dependent variable: yes/no) and cultural group (independent variable: using Myanmar/Thailand, the largest among all six groups, as the comparison group). Wald χ^2^ p-values less than 0.05 were considered statistically significant. All descriptive and statistical analyses were carried out using SAS® software.

The University of North Texas Health Science Center North Texas Regional Institutional Review Board approved all study protocols. Written consent forms were translated into 13 languages with on-going consultation with project bilingual staff and community advisors. These consents were field-tested and refined prior to use with members of each respective immigrant community. Key personnel obtained verbal and written consent from all participants. While all participants received and signed a written consent, the consent was also read to some participants who could not read by themselves due to low literacy levels. The community health workers encouraged questions during the consenting process as another strategy to check for comprehension.

## Results

Baseline demographic characteristics of 874 refugees enrolled in the BB Program between March 2014 and September 2018 are shown in Table 1, stratified by cultural groups. The majority (81%) of participants were female, and more than half (53%) were younger than 40 years of age. Two-thirds of participants reported living in the U.S. for less than five years. Most (81%) participants reported not speaking English well or not at all. Seventy-six percent never attended school or had less than 12 years of education; 65% reported that they were unemployed; and 51% were without health insurance at enrollment into the BB Program.

**Table 1:** Baseline demographic characteristics of Building Bridges program participants, by geographic origin, Fort Worth, Texas, March 2014 –September 2018 (N = 874).

Characteristic	Total	Myanmar^a^	Central African Region^b^	Bhutan^c^	Somalia^d^	Arabic Speaking Countries^e^	Others^f^
**# of Participants, n (%)**	**874 (100)**	285 (33)	194 (22)	163 (19)	141 (16)	67 (8)	24 (3)
**Gender**	**874 (100)**						
Female	704 (81)	240 (34)	150 (21)	116 (16)	116 (16)	60 (9)	22 (3)
Male	170 (19)	45 (26)	44 (26)	47 (28)	25 (15)	7 (4)	2 (1)
**Age (in years), n (%)**	**874 (100)**						
29 or below	233 (27)	97 (42)	45 (19)	15 (6)	60 (26)	7 (3)	9 (4)
30–39	223 (26)	78 (35)	46 (21)	32 (14)	31 (14)	27 (12)	9 (4)
40–49	207 (24)	59 (29)	51 (25)	43 (21)	28 (14)	23 (11)	3 (1)
50–59	111 (13)	26 (23)	24 (22)	41 (37)	14 (13)	5 (5)	1 (1)
60 or above	100 (11)	25 (25)	28 (28)	32 (32)	8 (8)	5 (5)	2 (2)
**Years lived in US, n (%)**	**874 (100)**						
Less than 5 years	575 (66)	158 (27)	144 (25)	123 (21)	82 (14)	50 (9)	18 (3)
5–10 years	249 (28)	123 (49)	39 (16)	35 (14)	37 (15)	9 (4)	6 (2)
More than 10 years	50 (6)	4 (8)	11 (22)	5 (10)	22 (44)	8 (16)	0 (0)
**Children, n (%)**	**866 (99)**						
Yes	705 (81)	245 (35)	133 (19)	146 (21)	105 (15)	59 (8)	17 (2)
No	161 (19)	38 (24)	57 (35)	17 (11)	36 (22)	6 (4)	7 (4)
**Number of Biological Children, n (%)**	**697 (99)**						
1–2	244 (35)	95 (39)	30 (12)	63 (26)	30 (12)	21 (9)	5 (2)
3–5	327 (47)	123 (38)	68 (21)	61 (19)	37 (11)	29 (9)	9 (3)
6+	126 (18)	26 (21)	32 (25)	20 (16)	37 (29)	8 (6)	3 (2)
**# of school years attended for formal education, n (%)**	**874 (100)**						
None	278 (32)	110 (40)	37 (13)	57 (21)	55 (20)	14 (5)	5 (2)
Less than 12 years	388 (44)	147 (38)	78 (20)	65 (17)	69 (18)	15 (4)	14 (4)
12 years or more	208 (24)	28 (13)	79 (38)	41 (20)	17 (8)	38 (18)	5 (2)
**How well do you speak English?, n (%)**	**798 (91)**						
Not at all/ Not well	648(81)	223 (34)	145 (22)	127 (20)	104 (16)	34 (5)	15 (2)
Well/Very well	150 (19)	52 (35)	30 (20)	28 (19)	15 (10)	20 (13)	5 (3)
**Currently Employed, n (%)**	**868 (99)**						
Yes	307 (35)	93 (30)	79 (26)	54 (18)	52 (17)	24 (8)	5 (2)
No	561 (65)	190 (34)	111 (20)	109 (20)	89 (16)	43 (8)	19 (3)
**Current Health Insurance, n (%)**	**865 (99)**						
Yes	422 (49)	131 (31)	94 (22)	87 (21)	75 (18)	24 (6)	11 (3)
No	443 (51)	149 (34)	97 (22)	76 (17)	66 (15)	42 (9)	13 (3)

^**a**^ Includes participants from both Myanmar (n = 282) and Thailand (n = 3).

^**b**^ Includes participants from: Congo (n = 124), Rwanda (n = 50), Burundi (n = 17), Tanzania (n = 1), Uganda (n = 1), and Kenya (n = 1, who reported Swahili as native language).

^**c**^ Includes participants from both Bhutan (n = 135) and Nepal (n = 28).

^**d**^ Includes participants from both Somalia (n = 138) and Kenya (n = 3, who reported Somali as their native language).

^**e**^ Includes participants from Arabic speaking countries: Sudan (n = 43), Iraq (n = 12), Jordan (n = 4), Syria (n = 7), and, Egypt (n = 1).

^**f**^ Includes participants from: Afghanistan (n = 3), Angola (n = 1), Chad (n = 4), Eritrea (n = 7), Ethiopia (n = 7), Liberia (n = 1), and, Senegal (n = 1).

**Fig 1:**
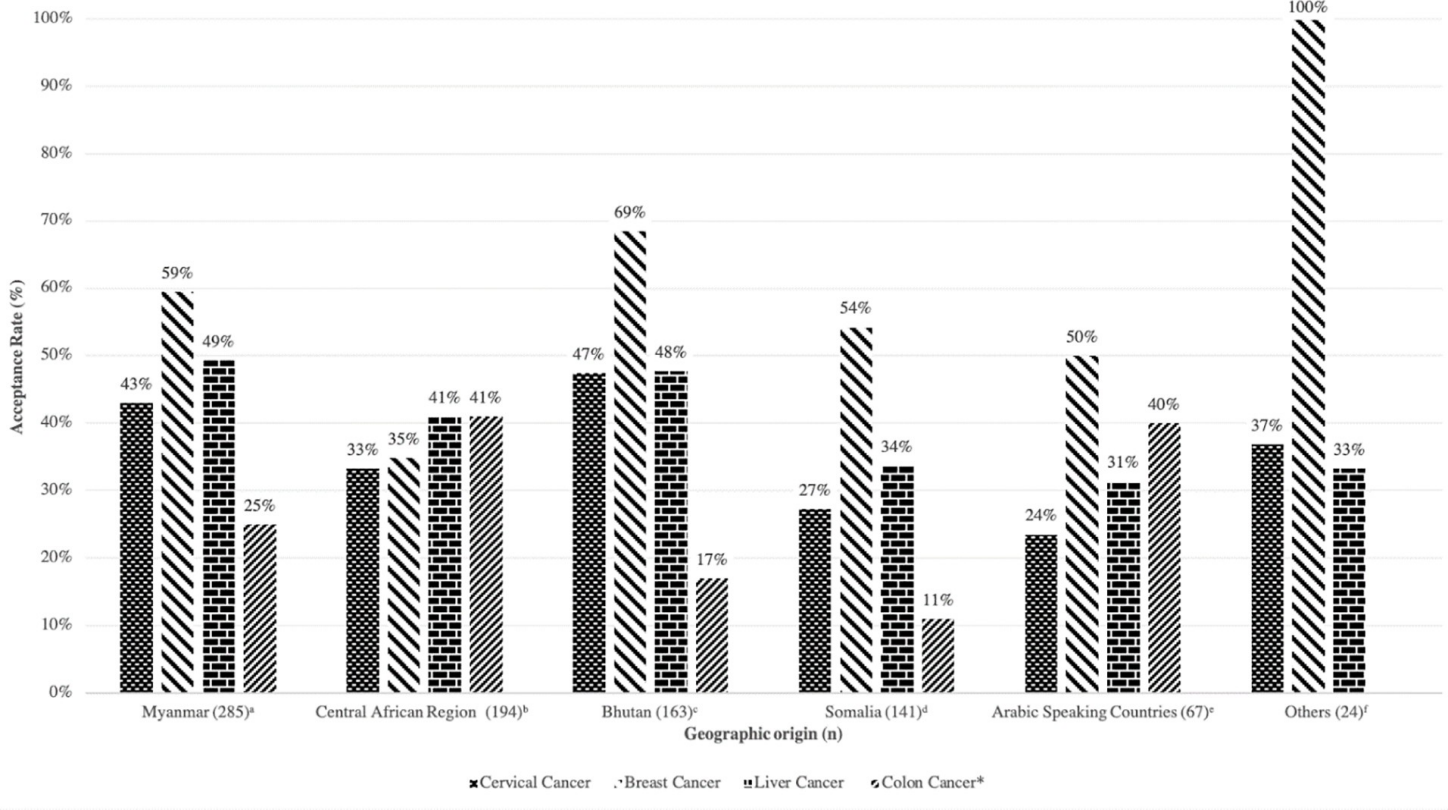
Utilization of cancer screenings among Building Bridges program participants, by geographic origin, Fort Worth, Texas, March 2014 –September 2018 (N = 874). *Colon cancer screening was offered to those enrolled after 1st March 2017 and met the age of eligibility. No participant from ‘Others’ group was eligible for colon cancer screening. ^a^ Includes participants from both Myanmar (n = 282) and Thailand (n = 3), ^b^ Includes participants from: Congo (n = 124), Rwanda (n = 50), Burundi (n = 17), Tanzania (n = 1), Uganda (n = 1), and Kenya (n = 1, who reported Swahili as native language), ^c^ Includes participants from both Bhutan (n = 135) and Nepal (n = 28), ^d^ Includes participants from both Somalia (n = 138) and Kenya (n = 3, who reported Somali as their native language), ^e^ Includes participants from Arabic speaking countries: Sudan (n = 43), Iraq (n = 12), Jordan (n = 4), Syria (n = 7), and, Egypt (n = 1), ^f^ Includes participants from: Afghanistan (n = 3), Angola (n = 1), Chad (n = 4), Eritrea (n = 7), Ethiopia (n = 7), Liberia(n = 1), and, Senegal (n = 1).

Overall uptake of screening services for each cancer type (cervical, breast, liver, and colon) among eligible participants by gender is shown in Table 2. The majority of participants screened were women. Among 662 age-eligible women across all cultural groups, 542 (82%) were eligible for a cervical cancer screen. Of those, 200 (37%) completed a cervical exam. Nearly three-quarters (74%) of those screened had never had a cervical cancer screen before. For cervical cancer screening, the Bhutanese had the highest (47%) uptake among all six groups (See Fig 1). Following Bhutan, Myanmar had the second highest (43%), and the Arabic group had the lowest (24%) uptake for cervical cancer screening among all groups.

**Table 2:** Utilization of cancer screening services among Building Bridges program participants by gender, March 2014 –September 2018 (N = 874)*.

Characteristic	Cervical Cancer (PAP Exam)	Breast Cancer (Mammogram)	Liver Cancer (Hepatitis B Screening)	Colon Cancer^a^ (FIT)
Eligible for Screening (among those who met the age of eligibility)^**b**^, N/D (%)	542/662 (82)	253/313 (81)	600/874 (69)	59/68 (87)
Female	542/662 (82)	253/313 (81)	484/704 (69)	40/48 (83)
Male	NA	NA	116/170 (68)	19/20 (95)
Never Screened Before (among those eligible), n (%)	358 (66)	184 (73)	583 (97)	59 (100)
Female	358 (66)	184 (73)	471 (97)	40 (100)
Male	NA	NA	112 (97)	19 (100)
Completed Screening, n (%)	200 (37)	136 (54)	265 (44)	16 (27)
Female	200 (37)	136 (54)	227 (47)	11 (28)
Male	NA	NA	38 (33)	5 (26)
Never Screened Before (among those screened), n (%)	147 (74)	107 (79)	257 (97)	16 (100)
Female	147 (74)	107 (79)	222 (98)	11 (100)
Male	NA	NA	35 (92)	5 (100)
Screening Results, n (%)				
Normal	169 (84)	104 (76)	229 (86)	9 (56)
Abnormal	8 (4)	15 (11)	11 (4)	0 (0)
Unknown/ Pending	23 (12)	17 (13)	25 (9)	7 (44)

Abbreviations: PAP, Papanicolaou test; FIT, Fecal Immunochemical Test; N, Numerator; D, Denominator; NA, Not Applicable.

^*^ N = 874 total participants, including 704 (81%) Female and 170 (19%) Males.

^**a**^ Colon cancer screening was offered to those enrolled after 1st March 2017 and met the age of eligibility.

^**b**^ as per standard guidelines, 21, 40, 18, and 50 were chosen as the age of eligibility for Cervical, Breast, Liver and Colon cancer screening, respectively.

For cervical and breast cancer screenings, we observed statistically significant associations between the cultural groups and screening uptake for each p-value = 0.01 (Table 3). Also, we found a statistically significant difference in utilization of cervical cancer screening for the Arabic group (OR = 0.41; 95% CI = 0.20, 0.83) and Somali group (OR = 0.50; 95% CI = 0.28, 0.87). No statistically significant difference in uptake of cervical cancer screening was observed for Central African region, Bhutanese, and Others when compared with Myanmar groups.

**Table 3:** Utilization of cancer screening services among Building Bridges program participants, by geographic origin, Fort Worth, Texas, March 2014 –September 2018 (N = 874).

Cancer Screening	Total	Myanmar^a^	Central African Region^b^	Bhutan^c^	Somalia^d^	Arabic Speaking Countries^e^	Others^f^
**Cervical (PAP)**							
Eligible for Screening (among age eligible), n (%)	542 (82)	181 (33)	129 (24)	78 (14)	84 (15)	51 (9)	19 (4)
Accepted screening	200 (37)	78 (43)	43 (33)	37 (47)	23 (27)	12 (24)	7 (37)
No Screening	342 (63)	103 (57)	86 (67)	41 (53)	61 (73)	39 (76)	12 (63)
Chi-Square p-value	0.01*	-	0.08	0.52	0.02*	0.01*	0.60
POR^**g**^ (95% CI)	-	-	0.66 (0.41, 1.06)	1.19 (0.70, 2.03)	0.50 (0.28, 0.87)	0.41 (0.20, 0.83)	0.77 (0.29, 2.05)
**Breast (Mammogram)**							
Eligible for Screening (among age eligible), n (%)	253 (81)	74 (29)	66 (26)	54 (21)	35 (14)	22 (9)	2 (1)
Accepted screening	136 (54)	44 (59)	23 (35)	37 (69)	19 (54)	11 (50)	2 (100)
No Screening	117 (46)	30 (41)	43 (65)	17 (31)	16 (46)	11 (50)	0 (0)
Chi-Square p-value	0.01*	-	0.01*	0.30	0.61	0.43	0.99
POR^**g**^ (95% CI)	-	-	0.37 (0.18, 0.73)	1.48 (0.71, 3.11)	0.81(0.36, 1.82)	0.68 (0.26, 1.77)	-
**Liver (Hepatitis B screen)**							
Eligible for Screening (among age eligible), n (%)	600 (69)	225 (38)	90 (15)	155 (26)	83 (14)	35 (6)	12 (2)
Accepted screening	265 (44)	111 (49)	37 (41)	74 (48)	28 (34)	11 (31)	4 (33)
No Screening	335 (56)	114 (51)	53 (59)	81 (52)	55 (66)	24 (69)	8 (67)
Chi-Square p-value	0.08	-	0.19	0.76	0.02*	0.05	0.29
POR^**g**^ (95% CI)	-	-	0.72 (0.44, 1.18)	0.94 (0.62, 1.41)	0.52 (0.31, 0.88)	0.47 (0.22, 1.01)	0.51 (0.15, 1.75)
**Colon (FIT)**							
Eligible for Screening (among age eligible), n (%)	59 (87)	16 (27)	17 (29)	12 (20)	9 (15)	5 (8)	0 (0)
Accepted screening	16 (27)	4 (25)	7 (41)	2 (17)	1 (11)	2 (40)	0 (0)
No Screening	43 (73)	12 (75)	10 (59)	10 (83)	8 (89)	3 (60)	0 (0)
Chi-Square p-value	0.44	-	0.33	0.60	0.42	0.52	-
POR^**g**^ (95% CI)	-	-	2.10 (0.47, 9.30)	0.60 (0.09, 3.99)	0.38 (0.04, 4.00)	2.00 (0.24, 16.61)	-

Abbreviations: PAP, Papanicolaou test; FIT, Fecal Immunochemical Test; NA, not applicable; POR, Prevalence Odds Ratio; CI, Confidence Interval.

^**a**^ Includes participants from both Myanmar (n = 282) and Thailand (n = 3),

^**b**^ Includes participants from: Congo (n = 124), Rwanda (n = 50), Burundi (n = 17), Tanzania (n = 1), Uganda (n = 1), and Kenya (n = 1, who reported Swahili as native language),

^**c**^ Includes participants from both Bhutan (n = 135) and Nepal (n = 28),

^**d**^ Includes participants from both Somalia (n = 138) and Kenya (n = 3, who reported Somali as their native language),

^**e**^ Includes participants from Arabic speaking countries: Sudan (n = 43), Iraq (n = 12), Jordan (n = 4), Syria (n = 7), and, Egypt (n = 1),

^**f**^ Includes participants from: Afghanistan (n = 3), Angola (n = 1), Chad (n = 4), Eritrea (n = 7), Ethiopia (n = 7), Liberia(n = 1), and, Senegal (n = 1),

^**g**^ Crude OR

*P < .05

The uptake for breast cancer screening was 50% or more in all cultural groups except one, Central African region (35%). The odds of participants from the Central African region group accepting a mammogram was 63% lower than the odds of accepting a mammogram from members of the Myanmar group (POR = 0.37, 95% CI = 0.18–0.73, p-value = 0.01). More women received a mammogram (54%) and HBV screening (47%) compared to cervical screens, even though most mammogram and HBV completers were first-time screens (79% and 98%, respectively).

Uptake of HBV screens among men (33%) were lower compared to women (47%). Over 90% of men and 90% of women screened for HBV reported never being screened for HBV. The screening uptake for HBV screen was highest (49%) in the Myanmar group and lowest (31%) among participants from Arabic-speaking countries. Overall, no statistically significant association was found between uptake of HBV screens and geographic origin (p = 0.08). However, the odds of HBV screening acceptance from the Somali group was 48% lower than odds of HBV screening acceptance from the Myanmar group (OR = 0.52, 95% CI = 0.31–0.88, p-value = 0.02).

Among all four cancer screenings, the overall uptake was lowest (27%) for colon cancer screening. Colon cancer screens were accepted almost equally between men and women (26% vs. 28%, respectively). All men and women screened for colorectal cancer were first-time screeners. Uptake for colon cancer screening was highest in the Central African region and Arabic speaking countries (41% vs. 40%, respectively) among all six groups. Overall, no statistically significant association was noted between uptake of colon cancer screening and geographic origin (p = 0.19), though numbers were small in these groups.

## Discussion

In this cross-sectional study, we investigated differences in uptake of preventive cancer screenings among multi ethnic immigrants participating in the Building Bridges program-a community-based intervention focused on cancer prevention screenings among refugees. Additionally, we assessed the uptake among those reporting to have never been screened. Overall, the results illustrate variation in the uptake of different types of screenings, between the different ethnic groups assessed and evidence of reaching the never before screened. To our knowledge this study provides the most comprehensive evidence of cancer screening uptake among multiple refugee groups enrolled in a community program. There is a limited evidence base on preventive screening uptake among immigrant groups, as well as, how to implement culturally and linguistically appropriate cancer screenings among diverse populations.

Our results show that cancer screening uptake varied among refugee group by type of screening. We found significant differences in cervical, breast, and liver screening completion across the six refugee groups served through the BB program. However, we did not find evidence of differences in colon screening uptake between these groups, although the numbers were too small for meaningful conclusions. Overall, the Myanmar and Bhutanese groups had higher screening completion compared to Somali, Central African, and Arabic speaking groups. These differences may be explained by longer time resettled in the U.S. by the Myanmar, but it fails to explain the Bhutanese group who were more recent to the U.S. Even though the Myanmar and Bhutan groups reported not to speak English well and reported more unemployment than other groups, their uptake was higher than other groups in the study. Mammograms were the most accepted cancer screen among most refugees (54%), with the exception of the Central African group (35%).

Previous research has also shown that refugee groups respond differently to preventive screenings. For example, Samuels et al. found Somalis were significantly less likely to be screened than Vietnamese and Cambodian. [24] Another study comparing Somali immigrants with other African immigrant groups found a significant difference in ever being screened for breast cancer, but not for pap screening. [25]

That our study found a lower cervical screening uptake among Somali women compared to other ethnic groups align with the findings of a randomized control trial pilot study conducted in Minnesota using the same methodological approach of targeting participants outside of a health system or medical home. In this study, only 19.4% Somali women accepted the cervical cancer screening [26] compared to 27% of Somali women in our study. Similar findings were observed in the Boston area where Somali women were found to be less likely to be screened for cervical cancer than Central African women (21.3% vs. 44.1%). [25]

A higher cervical screening proportion of 48.79% among Somali women, however, was found in a clinical chart review in a primary care setting in Minnesota.[21] Although direct comparison is complicated by methodological and setting differences, there is evidence that the uptake of cervical cancer among Somali women is lower than other refugee groups.

For the Arabic-speaking community, the screening uptake for cervical cancer (24%) found in our study was lower compared to other findings from studies conducted in the US [15,18,27] For example, a survey of religion-related factors among American Muslim women conducted by Padela et al. in partnership with the Council of Islamic Organizations of Greater Chicago found that 84% had obtained a pap test in their lifetime. Screening differences may be due to variations in study design, recruitment methods, education type (e.g. Individual education vs. group, clinical chart review, DVD), and health educator used in each study. The lower screening uptake observed in our study may also be due to a difference in method when calculating screening uptake. We excluded women who were up-to-date with their pap exam at the time of recruitment since our program focused on increasing screening to recommended guidelines. However, other studies among Arabic populations in the U.S. have mostly measured women who have ever received a Pap in their lifetime. [15, 18, 27, 28]

Breast cancer screening was higher in our study than what was observed in research conducted in Buffalo, New York (54% vs 35%).[29] This study bears similarities to ours because it included the same culture groups and focused on never screened and not up-to-date participants. The difference in completion proportions between the studies may be explained by the type of education and assistive services offered. Study participants in Buffalo, NY, received health education and one-on-one navigation from an experienced community health educator with the use of interpreters while participants in the Building Bridges program received transportation and language interpretation assistance for screening appointments in addition to culturally tailored health education and one-on-one navigation provided by an individual community health worker from the same cultural group. Having a health educator of the same ethnic group has been shown to reduce significant cultural, linguistic, and logistical barriers to health care. [30]

Like our study, several studies conducted in Canada, found associations between regions of origin and mammogram screening. [31,32] In one of these studies, Vahabi, et al, found that Asian immigrants were more likely to complete screenings (62%) than African immigrant communities (40%).[31] The screening completion proportion among the sub-Saharan African refugees to Canada was 46.6% in another study.[31] In our study, breast cancer screenings exceeded the Vahabi study overall (54%), but the Central African group had lower screening completion (35%) for breast cancer compared to Myanmar group (59%). In our study, the proportion of participants from the Central African region accepting breast cancer screening were lowest among all groups compared to Myanmar. Similarly, in a survey conducted in the Greater Boston and New Hampshire area, only 25% of Congolese women were up-to-date with their mammogram exam. [25]

Colorectal (Colonoscopy and FIT) education and screening started in March 2017. Among the 68 participants eligible for screening, 59 had never received a prior screening (87%). Due to this small sample size, we focused on the overall screening proportions of those in the program. Other studies reported higher proportions of patients screened at baseline. For example, 54% of immigrants and refugees reported a prior screening in Toronto, Canada and in a study of Somalians in Minnesota reported prior screening proportions of 38.5%. [21,32] Data for these studies came from their patient populations collected through focus groups and chart reviews. This study design may reveal higher screening proportions because the participants were current patients of the health system, thereby increasing their exposure to screening opportunities. As discussed, 51% of our population lacked health insurance and were not enrolled in a discounted health program at baseline. This high percentage of uninsured may explain how we were able to reach refugees that had never been screened prior to participation in the program.

Our results from the Building Bridges program suggest that this program is reaching those who report never being screened (66% pap screening; 73% mammogram; 97% hepatitis, 87% colon screen) Additionally, among the never screened, we observed a high percentage of screening completion (74% pap screening; 79% mammogram; 97% hepatitis; 100% colon screen). Other studies among never screened resulted in refugee women in Buffalo (NY) following a community-based educational program, 20 of 58 (34%) women received a mammogram who had never completed a mammogram screening before intervention. [29]

United States domestic screening requirements for arriving refugees mandate refugees to have Hepatitis B Virus (HBV) screening.[33] However, few of our participants recalled a prior testing, nor could they verify their HBV status at the time of enrollment (69% did not know their status). This is similar to other studies that have reported a lack of awareness of HBV and/or HBV status in the general population.[34] When offered an HBV screening, acceptance proportions were <50% in each cultural group. Overall, there were no significant differences among culture groups for HBV screenings. To our knowledge, our study is the first to explore prior HBV screening and awareness of status post arrival in a community setting versus a clinic setting.

Some of these findings are not surprising and suggest that although while not simple to do, the act of making cancer screening outreach, education and screenings available in multiple languages and adapted to different cultures, does not automatically lead to adoption by the targeted communities.

Previous research shows the challenges of providing culturally and linguistically appropriate education to immigrant populations.[11–14] Despite the program removing most known barriers, screening uptake was lower in all populations compared to the U.S. national average and fell short of the Healthy People 2020 Targets of 93% for cervical, 81.1% for breast, and 70.5 for colorectal screening.[35] As of 2015, the screening proportions in the overall U.S. population were 81.2% for pap test, 71.6% for mammogram, and 62.4% for colon screening.[35] The low uptake of screening observed in our study are similar to findings from other studies reporting lower screening proportions among refugees and immigrants when compared to U.S. born populations.[19–21, 36] Acceptance of screening was highest among the Myanmar and Bhutanese groups. The Building Bridges program was most effective in these two culture groups. More research is needed to help explain not only the variation in uptake of cancer type, but also reasons for not screening in general. Additionally, given the substantial financial and staff investment in community health worker models, studies comparing the cost-effectiveness of models, i.e., community vs clinic based, is warranted. As efforts to include diverse immigrant populations expand, variation in uptake by type and population groups should be considered. The results of this analysis provide evidence that uptake in screenings vary among ethnic groups, even with a culturally and linguistically appropriate outreach and education strategy.

### Limitations

Many refugees have experienced significant trauma in their lives, which could affect their memory[37] and lead to recall bias during the baseline assessment. Additionally, except for a mammogram, cancer screenings can be easily confused with other types of medical procedures and screenings that require blood or stool samples. Trauma, health literacy, education levels, provider and system factors and communication are all possible contributors to a general lack of awareness of prior procedures among this population. Our uptake results were among those not up-to-date with screening, who reported never being screened, or did not know their prior screening history. Therefore, we excluded those who were receiving screenings in clinic settings. However, the purpose of the project was to reach individuals at higher risk of being under-screened, i.e., uninsured or not enrolled in the county discounted health care system. Describing the screening procedures during the assessment and including “I don’t know” as a response category helped to minimize recall bias.

The Texas health care environment may limit the generalizability of study findings to refugee populations in other states and counties. Unlike other common refugee study locations, such as Minnesota, Rochester (New York), Ontario, Massachusetts, and California, restrictive Medicaid policies and the lack of immigrant health outreach programs in Texas creates barriers to health care coverage and access to services post-resettlement.

Baseline assessment data (e.g., screening history) was self-reported. Characteristics of the community health workers (e.g., connections in the community, age, background, etc.) and cultural groups (e.g., internal diversity and inter-ethnic conflict) may have affected the willingness of community members to enroll and subsequently accept cancer screenings. A smaller sample size in the Arabic speaking group reflects delayed outreach into this population. We included the Arabic speaking population and colon cancer screening as part of a Cancer Prevention Research Institute of Texas (CPRIT) grant expansion beginning in March 2017. All other communities were included in the study starting from 2014. Our sample size is based on one year of enrollments and screenings offered through September 2018.

### Public health implications

Even though refugee admissions to the U.S. have steadily declined since 2016, [38] the need for culturally and linguistically appropriate outreach and education to diverse immigrant groups remains if we are to meet national goals, detect cancer early, save lives and reduce costs. Refugee and immigrant populations represent several of the key priority populations outlined by the Texas Cancer Plan because of their low education attainment, poverty, unavailable health insurance coverage and access, and health literacy challenges. Cancer Plans are “a blueprint for cancer research, prevention, and control in areas including risk reduction, early detection, and screening diagnosis, treatment, palliation, quality of life, survivorship, and production development”. Accordingly, the Texas Cancer Plan aims “to reduce the cancer burden across the state and improve the lives of Texans.” [39] As our study shows that while trauma, forced migration, and resettlement are shared experiences, populations differ in their willingness to accept cancer screenings.

Additionally, even when we removed or addressed known barriers (i.e., transportation, interpretation, scheduling, and appointment reminders), screening completion did not reach Healthy People 2020 targets for any group or screening type. Future studies should focus on alternate education delivery methods, such as online or video based, and within different study settings such as clinics and community locations. These approaches may help increase screening while reducing program costs. More work is needed to better identify the reasons participants chose not to participate in screenings. Failing to recognize the diversity within refugee communities and adapting to their specific cultural and linguistic needs with outreach, education, and information, will leave a substantial population of new Americans out of cancer elimination efforts.

## References

[1] Ratha D, Plaza S, Dervisevic E. Migration and remittances Factbook 2016. 3rd ed Washington, DC: The World Bank; 2016.

[2] Department USS. Refugee Admissions.: Bureau of Population, Refugees, and Migration.; [Available from: https://www.state.gov/j/prm/ra/.

[3] Department USS. Fact Sheet: Fiscal Year 2016 Refugee Admissions: Bureau of Population, Refugees, and Migration.; 2017 [Available from: https://www.state.gov/j/prm/releases/factsheets/2017/266365.htm.

[4] Bishehsari F, Mahdavinia M, Vacca M, Malekzadeh R, Mariani-Costantini R. Epidemiological transition of colorectal cancer in developing countries: environmental factors, molecular pathways, and opportunities for prevention. World journal of gastroenterology: WJG. 2014;20(20):6055 10.3748/wjg.v20.i20.6055 24876728 PMC4033445

[5] Franceschi S, Wild CP. Meeting the global demands of epidemiologic transition–The indispensable role of cancer prevention. Molecular oncology. 2013;7(1):1–13. 10.1016/j.molonc.2012.10.010 23218182 PMC5528406

[6] Ginsburg O, Bray F, Coleman MP, Vanderpuye V, Eniu A, Kotha SR, et al The global burden of women’s cancers: a grand challenge in global health. The Lancet. 2017;389(10071):847–60. 10.1016/S0140-6736(16)31392-7 PMC6191029 27814965

[7] Organization WH. Addressing the challenge of women's health in Africa: report of the Commission on Women's Health in the African Region: World Health Organization; 2012.

[8] Akinyemiju TF. Socio-economic and health access determinants of breast and cervical cancer screening in low-income countries: analysis of the World Health Survey. PloS one. 2012;7(11):e48834 10.1371/journal.pone.0048834 23155413 PMC3498259

[9] USA for UNHCR—The UN Refugee Agency. What is a Refugee? Definition and Meaning: USA for UNHCR: USA for UNHCR; 2019 [10/2/2019]. Available from: https://www.unrefugees.org/refugee-facts/what-is-a-refugee/.

[10] Anaman JA, Correa-Velez I, King J. Knowledge Adequacy on Cervical Cancer Among African Refugee and Non-Refugee Women in Brisbane, Australia. Journal of cancer education: the official journal of the American Association for Cancer Education. 2018;33(3):716–23. 27796876 10.1007/s13187-016-1126-y

[11] Haworth RJ, Margalit R, Ross C, Nepal T, Soliman AS. Knowledge, attitudes, and practices for cervical cancer screening among the Bhutanese refugee community in Omaha, Nebraska. Journal of community health. 2014;39(5):872–8. 10.1007/s10900-014-9906-y 25060231 PMC4175018

[12] Kue J, Hanegan H, Tan A. Perceptions of Cervical Cancer Screening, Screening Behavior, and Post-Migration Living Difficulties Among Bhutanese–Nepali Refugee Women in the United States. Journal of community health. 2017;42(6):1079–89. 10.1007/s10900-017-0355-2 28455671 PMC7008456

[13] Saadi A, Bond B, Percac-Lima S. Perspectives on preventive health care and barriers to breast cancer screening among Iraqi women refugees. Journal of immigrant and minority health. 2012;14(4):633–9. 10.1007/s10903-011-9520-3 21901446

[14] Saadi A, Bond BE, Percac-Lima S. Bosnian, Iraqi, and Somali refugee women speak: a comparative qualitative study of refugee health beliefs on preventive health and breast cancer screening. Women's Health Issues. 2015;25(5):501–8. 10.1016/j.whi.2015.06.005 26219676

[15] Salman KF. Health beliefs and practices related to cancer screening among Arab Muslim women in an urban community. Health care for women international. 2012;33(1):45–74. 10.1080/07399332.2011.610536 22150266

[16] Schuster RC, Rodriguez EM, Blosser M, Mongo A, Delvecchio-Hitchcock N, Kahn L, et al “They were just waiting to die”: Somali Bantu and Karen Experiences with Cancer Screening Pre-and Post-Resettlement in Buffalo, NY Journal of the National Medical Association 2018. 10.1016/j.jnma.2018.10.006 PMC8862611 30420078

[17] Zhang Y, Ornelas IJ, Do HH, Magarati M, Jackson JC, Taylor VM. Provider perspectives on promoting cervical cancer screening among refugee women. Journal of community health. 2017;42(3):583–90. 10.1007/s10900-016-0292-5 27838808 PMC5409861

[18] Dallo FJ, Kindratt TB. Disparities in vaccinations and cancer screening among US-and foreign-born Arab and European American non-Hispanic White women. Women's Health Issues. 2015;25(1):56–62. 10.1016/j.whi.2014.10.002 25498764

[19] Endeshaw M, Clarke T, Senkomago V, Saraiya M. Cervical cancer screening among women by birthplace and percent of lifetime living in the United States. Journal of lower genital tract disease. 2018;22(4):280–7. 10.1097/LGT.0000000000000422 30063576 PMC6664302

[20] Hall IJ, Tangka FK, Sabatino SA, Thompson TD, Graubard BI, Breen N. Peer Reviewed: Patterns and Trends in Cancer Screening in the United States. Preventing chronic disease. 2018;15. 10.5888/pcd15.170465 PMC6093265 30048233

[21] Morrison TB, Wieland ML, Cha SS, Rahman AS, Chaudhry R. Disparities in preventive health services among Somali immigrants and refugees. Journal of immigrant and minority health. 2012;14(6):968–74. 10.1007/s10903-012-9632-4 22585311

[22] National Cancer Institute's Research-Tested Intervention Programs. (2019, November 21). Retrieved February 5, 2020, from https://rtips.cancer.gov/rtips/index.do

[23] Force TUSPST. Published Recommendations. U.S. Preventive Services Task Force Internet: The U.S. Preventive Services Task Force; 2019 [updated October 2019; cited 2019 2 October]. Available from: https://www.uspreventiveservicestaskforce.org/BrowseRec/Index.

[24] Samuel PS, Pringle JP, James NW, Fielding SJ, Fairfield KM. Breast, cervical, and colorectal cancer screening rates amongst female Cambodian, Somali, and Vietnamese immigrants in the USA. International Journal for Equity in Health. 2009;8(1):30. 19682356 10.1186/1475-9276-8-30 PMC2731767

[25] Harcourt N, Ghebre RG, Whembolua G-L, Zhang Y, Osman SW, Okuyemi KS. Factors associated with breast and cervical cancer screening behavior among African immigrant women in Minnesota. Journal of immigrant and minority health. 2014;16(3):450–6. 10.1007/s10903-012-9766-4 23334709 PMC3644538

[26] Sewali B, Okuyemi KS, Askhir A, Belinson J, Vogel RI, Joseph A, et al Cervical cancer screening with clinic‐based Pap test versus home HPV test among Somali immigrant women in Minnesota: a pilot randomized controlled trial. Cancer medicine. 2015;4(4):620–31. 10.1002/cam4.429 25653188 PMC4402076

[27] Padela AI, Peek M, Johnson-Agbakwu CE, Hosseinian Z, Curlin F. Associations between religion-related factors and cervical cancer screening among Muslims in greater Chicago. Journal of lower genital tract disease. 2014;18(4):326 10.1097/LGT.0000000000000026 24914883 PMC4530503

[28] Darwish-Yassine M, Wing D. Cancer epidemiology in Arab Americans and Arabs outside the Middle East. Ethnicity & disease. 2005;15(1 Suppl 1):S1–5-8. 15787031

[29] Gondek M, Shogan M, Saad-Harfouche FG, Rodriguez EM, Erwin DO, Griswold K, et al Engaging immigrant and refugee women in breast health education. Journal of Cancer Education. 2015;30(3):593–8. 10.1007/s13187-014-0751-6 25385693 PMC4745125

[30] Miyamoto R. E. S., Hermosura A. H., & Miguel Acido D. A. R. (2019). A Culture-Based Family-Centered Health Navigation Intervention for Chronic Disease Management in Native Hawaiians. *Hawaii J Med Public Health*, 78–82. Retrieved from https://www.ncbi.nlm.nih.gov/pmc/articles/PMC6603898/ 31285975 PMC6603898

[31] Vahabi M, Lofters A, Kumar M, Glazier RH. Breast cancer screening disparities among immigrant women by world region of origin: a population‐based study in Ontario, Canada. Cancer medicine. 2016;5(7):1670–86. 10.1002/cam4.700 27105926 PMC4944895

[32] Wang A, Yung E, Nitti N, Shakya Y, Alamgir A, Lofters A. Breast and Colorectal Cancer Screening Barriers Among Immigrants and Refugees: A Mixed-Methods Study at Three Community Health Centres in Toronto, Canada. Journal of immigrant and minority health. 2018:1–10. 10.1007/s10903-017-0556-x 29968004

[33] CDC. Guidelines for pre-departure and post-arrival medical screening and treatment of U.S.-bound refugees Centers for Disease Control and Prevention website: U.S. Department of Health & Human Services; [updated March 29, 2012. Available from: https://www.cdc.gov/immigrantrefugeehealth/guidelines/refugee-guidelines.html.

[34] Colvin, H. M., & Mitchell, A. E. (2010). Hepatitis and Liver Cancer: A National Strategy for Prevention and Control of Hepatitis B and C. *Committee on Prevention and Control of Viral Hepatitis Infections* Retrieved from http://citeseerx.ist.psu.edu/viewdoc/download?doi=10.1.1.173.5842&rep=rep1&type=pdf 25032367

[35] (ODPHP) OoDPaHP. Healthy People 2020 Objectives—Targeted proportion of adults receiving Carvical, Breast, Livar and Colorectal cancer screening based on the most recent guidelines The U.S. Department of Health and Human Services; 2008 [updated 4/2/19; cited Available from: https://www.healthypeople.gov/2020/topics-objectives/topic/cancer/objectives.

[36] Forney-Gorman A, Kozhimannil KB. Differences in cervical cancer screening between African-American versus African-born black women in the United States. Journal of immigrant and minority health. 2016;18(6):1371–7. 10.1007/s10903-015-0267-0 26349483

[37] Apitzsch H. (2009). Trauma and Dissociation in Refugee Patients. *Nordic Journal of Psychiatry*, 50(4), 333–336. 10.3109/08039489609078175

[38] Batalova BBaJ. Refugees and Asylees in the United States Internet: Migration Policy Institute; 2019 [updated June 13, 2019; cited 2019 2 October]. Available from: https://www.migrationpolicy.org/article/refugees-and-asylees-united-states.

[39] Texas CPaRIo, (CPRIT). 2018 Texas Cancer Plan. 2018.

